# The noradrenergic profile of plasma metanephrine in neuroblastoma patients is reproduced in xenograft mice models and arise from PNMT downregulation

**DOI:** 10.18632/oncotarget.27858

**Published:** 2021-01-05

**Authors:** Karim Abid, Maja Beck Popovic, Katia Balmas Bourloud, Jacqueline Schoumans, Joana Grand-Guillaume, Eric Grouzmann, Annick Mühlethaler-Mottet

**Affiliations:** ^1^Catecholamine and Peptides Laboratory, Service of Clinical Pharmacology and Toxicology, Lausanne University Hospital and University of Lausanne, Switzerland; ^2^Pediatric Hematology-Oncology Unit, Woman-Mother-Child Department, Lausanne University Hospital and University of Lausanne, Switzerland; ^3^Pediatric Hematology-Oncology Research Laboratory, Woman-Mother-Child Department, Lausanne University Hospital and University of Lausanne, Switzerland; ^4^Oncogenomics Laboratory, Hematology Service, Laboratory Medicine and Pathology Department, Lausanne University Hospital and University of Lausanne, Switzerland

**Keywords:** neuroblastoma, metanephrine, patient-derived xenograft, mouse model, Phenylethanolamine N-Methyltransferase

## Abstract

Metanephrines (MNs; normetanephrine (NMN), metanephrine (MN) and methoxytyramine (MT)) detected in urine or plasma represent the best biomarker for neuroblastoma (NB) diagnosis, however the metabolism of both catecholamine (CAT) and MNs remains enigmatic in NB. Using patient-derived xenograft (PDX) models derived from primary NB cells, we observed that the plasma levels of MNs in NB-PDX-bearing mice were comparable as in patients. Interestingly, murine plasma displayed an elevated fraction of glucuronidated forms of MNs relative to human plasma where sulfonated forms prevail. In tumors, the concentration ranges of MNs and CAT and the expression levels of the main genes involved in catecholamine metabolism were similar between NB-PDX and human NB tissues. Likewise, plasma and intratumoral profiles of individual MNs, with increased levels of MT and NMN relative to MN, were also conserved in mouse models as in patients. We further demonstrated the downregulation of the Phenylethanolamine N-Methyltransferase gene in NB biopsies and in NB-PDX explaining this biochemical phenotype, and giving a rational to the low levels of epinephrine and MN measured in NB affected patients. Thus, our subcutaneous murine NB-PDX models not only reproduce the phenotype of primary NB tumors, but also the metabolism of catecholamine as observed in patients. This may potentially open new avenues in preclinical studies for the follow up of novel therapeutic options for NB through the quantification of plasma MNs.

## INTRODUCTION

NB is an embryonic tumor of the sympathetic nervous system, that can arise at any site of the sympathetic chain but most frequently in the abdominal region [1, 2]. NB represents approximately 5% of all childhood malignancies, but accounts for 12% of childhood cancer-related mortality [3]. Metastases frequently occur in NB with an incidence of around 70% of cases [4] and 50 to 60% of high-risk NB will display relapse [2].

Catecholamine (CAT; E: epinephrine, NE: norepinephrine and DA: dopamine) production takes mainly place in adrenal chromaffin cells and sympathetic nerves. CAT are synthesized from L-tyrosine, which originates from diet and from hydroxylation of phenylalanine in the liver. L-tyrosine is transformed into dihydroxyphenylalanine (DOPA) by the cytoplasmic enzyme tyrosine hydroxylase (TH). DOPA is then transformed in the cytoplasm into DA by the enzyme aromatic L-amino acid decarboxylase (AADC) [5]. In chromaffin cells, DA is internalized into neurosecretory vesicles by the vesicular monoamine transporters (VMAT1, and VMAT2, respectively SLC18A1 and SLC18A2) where it is converted into NE by dopamine beta-hydroxylase (DBH) and NE could be further converted into E in neurosecretory vesicles containing Phenylethanolamine N-Methyltransferase (PNMT) [5]. Following sympathetic stimulation, CAT stored in vesicles are exocyted in the blood stream to reach their target receptors and initiate the classically described “fight or flight response” [6]. A small proportion of CAT that leak from neurosecretory vesicles is transformed into metanephrines (MNs; normetanephrine (NMN), metanephrine (MN) and methoxytyramine (MT)) by the enzyme COMT (catechol O-methyltransferase) [5]. MNs diffuse freely across the membrane and are released in the bloodstream. A large amount of MNs is transformed through multiple steps involving other intermediate products into vanillylmandelic acid (VMA, from MN and NMN) and homovanillic acid (HVA from MT). These two end-stage are then filtered by kidneys to be secreted in urine [5, 7, 8]. Studies involving the enzymes responsible for CAT synthesis in NB compared with healthy adrenal medulla have been scarce, with the majority of studies focusing on pheochromocytoma and paraganglioma (PHEO/PGL) two other neuroendocrine tumors secreting MNs. CAT metabolism in PHEO/PGL has been extensively reviewed elsewhere [5, 8]. The large amount of CAT found in tumors and in plasma has been shown to be a consequence of the over-expression of several enzymes involved in CAT synthesis [9, 10].

The biological bases of CAT and MNs metabolism remain enigmatic in patients affected by NB. NMN and especially MT have recently been described as new biomarker of NB in plasma and urine [11, 12] with better sensibility and sensitivity compared with CAT and VMA and HVA, the classical metabolites used for NB screening in urine of suspected patient. High MNs concentration in plasma arise from a high synthesis of CAT [5, 8] and are suspected to directly arise from NB, as extensively described for PHEO/PGL. However, NB biopsy availability is a limitation, precluding large-scale molecular studies to decipher CAT metabolism in these tumors. In this study, we validated the use of murine NB patient-derived xenografts (PDX) as a pertinent model to mimic NB from patients and we demonstrated that the noradrenergic phenotype of NB is due to PNMT downregulation.

## RESULTS

### Murine MNs are mainly found as glucuro conjugates forms

In this study, we used PDX derived from seven high-risk stage M NB patients (Table 1). Four NB-PDX models were previously published by our group [13]. In addition, we developed four novel NB-PDX models from either NB cells disseminated in the bone marrow (BM) (for three patients NB11, NB13, NB14) or from the primary tumor of the NB-1 patient (from which a NB-PDX derived from BM metastatic cells was previously described [13]) (Table 1). To validate our novel NB-PDX models, the histological phenotype and the genomic profiles of the xenografts were analyzed. H&E and IHC staining for Phox2b, TH, DBH, synaptophysin (SYP), and CD56 confirmed that our novel NB-PDX displayed a histological phenotype comparable to NB primary tumors and previously published NB-PDX (Supplementary Figure 1A). In addition, examination of genomic copy number alterations (CNA) by single-nucleotide polymorphism (SNP) array of the novel NB-PDX indicated that the overall genomic profile of segmental chromosomal alterations (SCA) was conserved between the primary tumor and the PDX generated from the corresponding patient, although several additional SCA were observed in 3 out of 4 PDX (Supplementary Table 1). These data indicated that genetically and phenotypically our NB-PDX mimic human NB and may represent relevant *in vivo* models for the study of CAT metabolism in NB.

**Table 1:** Clinical and biologic characteristics of NB samples and indication of the analyses carried out for each NB-PDX in this study

Primary tumors						NB-PDX		Analyses performed with NB-PDX					
Patient	Sex	Age at diagnosis	Risk group	Stage	MYCN status	Name	Source	IHC	genomic profile	CAT/MNs plasma	CAT/MNs tumor	mRNA for CAT genes	prot PNMT
NB1^*^	M	10 mo	HR	M	no amp	NB1-T-1	primary tumor	yes	yes	yes	yes	yes	yes
						NB1-BM-5	BM (diagn)^*^	yes	n.d.	n.d.^**^	yes	yes	n.d.
NB2^*^	F	44 mo	HR	M	no amp	NB2-BM-4	BM (diagn)^*^	yes	n.d.	n.d.^**^	yes	yes	n.d.
NB4^*^	M	143 mo	HR	M	no amp	NB4-BM-8	BM (relapse)^*^	yes	n.d.	yes	yes	n.d.	yes
NB5^*^	M	24 mo	HR	M	amp	NB5-BM-1	BM (diagn)^*^	yes	n.d.	n.d.^**^	yes	yes	n.d.
**NB11**	M	28 mo	HR	M	amp	NB11-BM-1	BM (diagn)	yes	yes	yes	yes	yes	yes
**NB13**	M	33 mo	HR	M	amp	NB13-BM-1	BM (diagn)	yes	yes	yes	yes	yes	yes
**NB14**	F	48 mo	HR	M	amp	NB14-BM-1	BM (diagn)	yes	yes	yes	yes	yes	yes

NB1 to NB14 correspond to NB patients. T, tumor; BM, bone marrow; F, female; M, male; mo, month; HR, high–risk group; amp, amplified MYCN locus; no amp, no amplification of MYCN locus, n.d., not done. NB cells were isolated from primary tumor (-T) or bone marrow aspirations (-BM) and cultured *in vitro* for few passages before subcutaneous injection into athymic Swiss nude mice. The number of *in vivo* passages in PDX are indicated at the end of the PDX name. ^*^Published in [13]; ^**^CAT/MNs evaluation in plasma not performed, as the plasma were not collected from these mouse models at the time at which we sacrificed the mice for the study described in [13].

**Figure 1:**
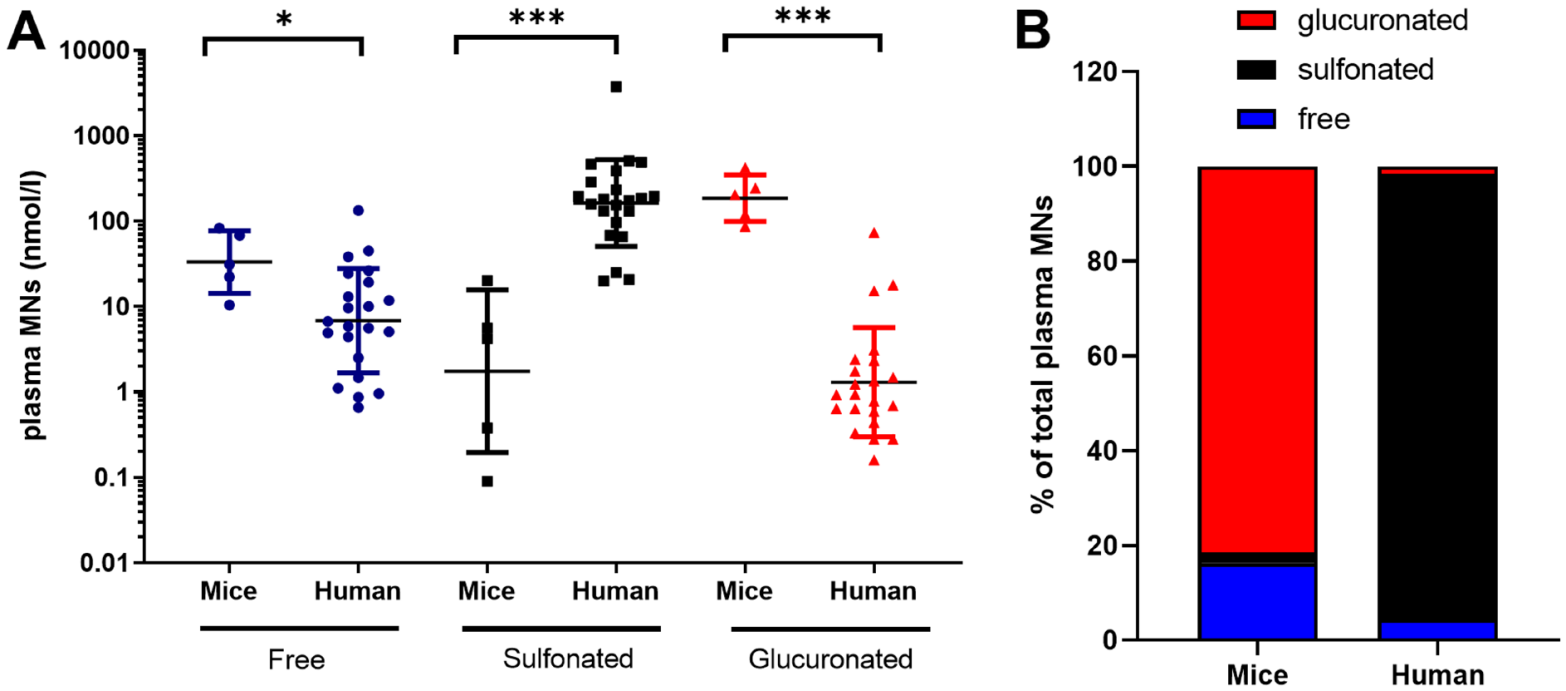
Free, sulfonated and glucuronidated forms of MNs (MT+NMN+MN) in plasma of mice with NB-PDX (*n* = 5) and NB patients (*n* = 22). (**A**) Values and geo mean ± geo SD are plotted on a logarithmic scale and analyzed with a non-parametric Mann Whitney test ^***^ *p* ≤ 0.0001, ^*^ *p* = 0.019. Geo. mean in mice and human were respectively as follow: free MNs: 33.3 and 6.8 nmole/l, sulfonated MNs: 1.7 and 162.8 nmole/l, and glucuronidated MNs: 185.8 and 1.3 nmole/l; (**B**) The relative amount of free, sulfonated and glucuronidated forms of MNs shown in (a) were plotted in the graph as percent of total plasma MNs (MT+NMN+MN).

We then investigated and compared the concentration of MNs in the plasma of NB patients and NB-PDX-bearing mice. We first measured the amounts of free, sulfonated and glucuronidated forms of plasma MNs as conjugation (sulfonation or glucuronidation) represents a major pathway for CAT and MNs inactivation and besides enabling renal clearance [14]. We observed in patients with NB (*n* = 22) a large proportion of sulfonated MNs compared with free and glucuronidated forms (Figure 1A), corresponding respectively to 94.1%, 4.4% and 1.5% of total MNs (sum of free, sulfonated and glucuronidated forms) (Figure 1B). This contrasts with MNs in murine plasma (*n* = 5) where MNs are found in majority as glucuronidated forms (Figure 1A), representing 81.5% of total MNs, while free MNs forms were less abundant (16.2%), and an even smaller proportion of sulfonated forms 2.3% was detected (Figure 1B). Considering the large differences in sulfonation and glucuronation, we thereafter quantified plasma total MNs in mice as the sum of free, sulfonated and glucuronidated forms. For the human samples, as plasma MNs concentrations described in this study for controls represent clinical values which lack measurement of glucuronidated forms of MNs, glucuronidated forms of MNs showed in Figure 1 were subsequently omitted for NB patients to ensure relevant comparisons of total MN, NMN and MT levels between patients and controls. This has however no impact as glucuronidated forms represent only 1.5% of total MNs in plasma. It is noteworthy that quantification of MNs in tissue represents free forms since no conjugated forms are produced within tumor tissues or control adrenal glands as described in PHEO/PGL ([5] and from our own observation during MNs quantification in NB tumors from patients and NB-PDX).

### MT and NMN represent NB biomarkers in the plasma of NB-PDX-bearing mice similarly to NB patients

**Figure 2:**
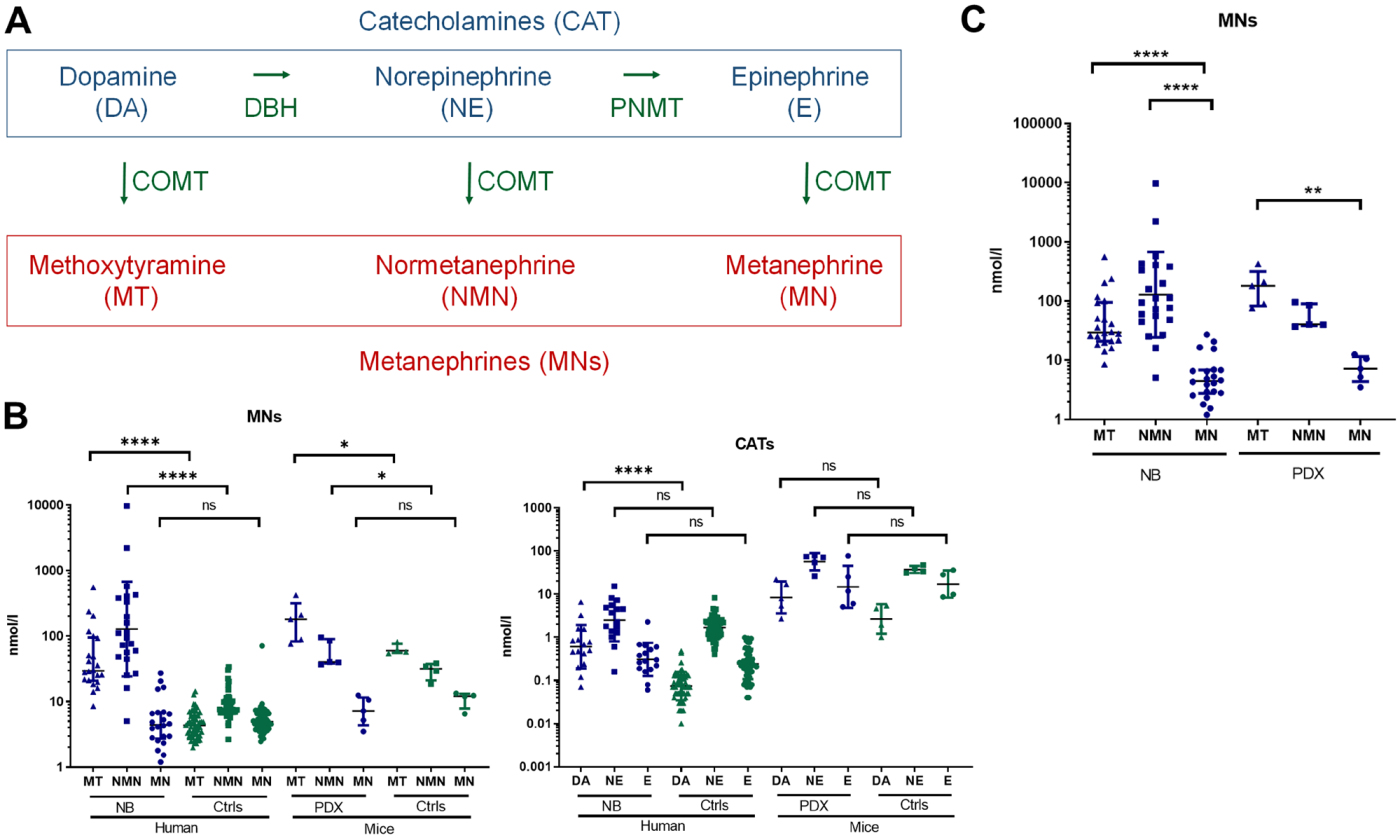
(**A**) Simplified scheme illustrating CAT and MNs metabolite biosynthesis. (**B**) Total plasma MNs (left panel) and CAT (right panel) in patients with NB (*n* = 22) compared with controls (*n* = 55) and in mice with NB-PDX (*n* = 5) compared with control mice (*n* = 4). Geo. mean for human samples in nmol/l for respectively MN, NMN and MT in NB patients: 4.83, 127.86 and 42.14; and for human controls: 5.17, 8.69 and 4.44. Mann Whitney test: ^***^ *p* ≤ 0.0001 for both NMN and MT (tumors compared with controls). PDX MNs levels are reported in the text, ^*^ *p* ≤ 0.05. (**C**) Illustration of total plasma MNs in patients NB (*n* = 22) and PDX NB mice (*n* = 5), value similar as in (B), to compare the relative amount each individual MNs. Kruskal-Wallis multiple comparison test: ^****^ *p* ≤ 0.0001, ^**^ *p* = 0.0027.

Then we assessed whether our NB-PDX models were mimicking NB in patients regarding MNs concentration and profile in plasma. To note CAT (DA, NE and E in the order of synthesis) are transformed by COMT into MT, NMN and MN, respectively (Figure 2A) and NB are characterized by an elevated amount of MT and NMN in plasma and urine [5, 15]. As observed in patients affected by a NB compared to healthy human subject, MT and NMN plasma concentrations in PDX mice were significantly increased compared with healthy mice (geo. mean at 161.9 nmol/l vs 63.2 nmol/l and 55.4 nmol/l vs 29.1 nmol/l respectively, ^*^ *p* = 0.0317 for both comparison) (Figure 2B, left panel). As expected, MN concentration was not significantly different between NB-PDX-bearing mice and controls (7.1 nmol/l vs 10.6 nmol/l) (Figure 2B, left panel). Even though plasma CAT concentration does not represent a reliable biomarker for the presence of a NB, DA is clearly increased in PDX mice compared with control (8.4 nmol/l vs 2.6 nmol/l, but without reaching significance threshold *p* = 0.07), whereas no significant differences for E (14.8 nmol/l vs 17.2 nmol/l) were measured; however a trend was also observed for NE: 56.7 nmol/l vs 37.3 nmol/l *p* = 0.2 (Figure 2B, right panel). This is in agreement with CAT levels quantified in patients (Figure 2B, right panel). Interestingly, a deeper analysis of the plasma MNs profile in human and mice revealed that MN level is very low compared to MT and NMN both in NB patients (geo mean 4.83 nmol/l vs 42.14 and 127.9 and nmol/l, respectively) and NB-PDX-bearing mice (geo mean 7.05 nmol/l vs 160.8 and 54.30 nmol/l, respectively) (Figure 2C). This is consistent with the fact that both MT and NMN represent biomarkers of NB in both urine and plasma [11, 12].

### NB-PDX and human primary NB displayed no differences for CAT and MNs tissue concentration

**Figure 3:**
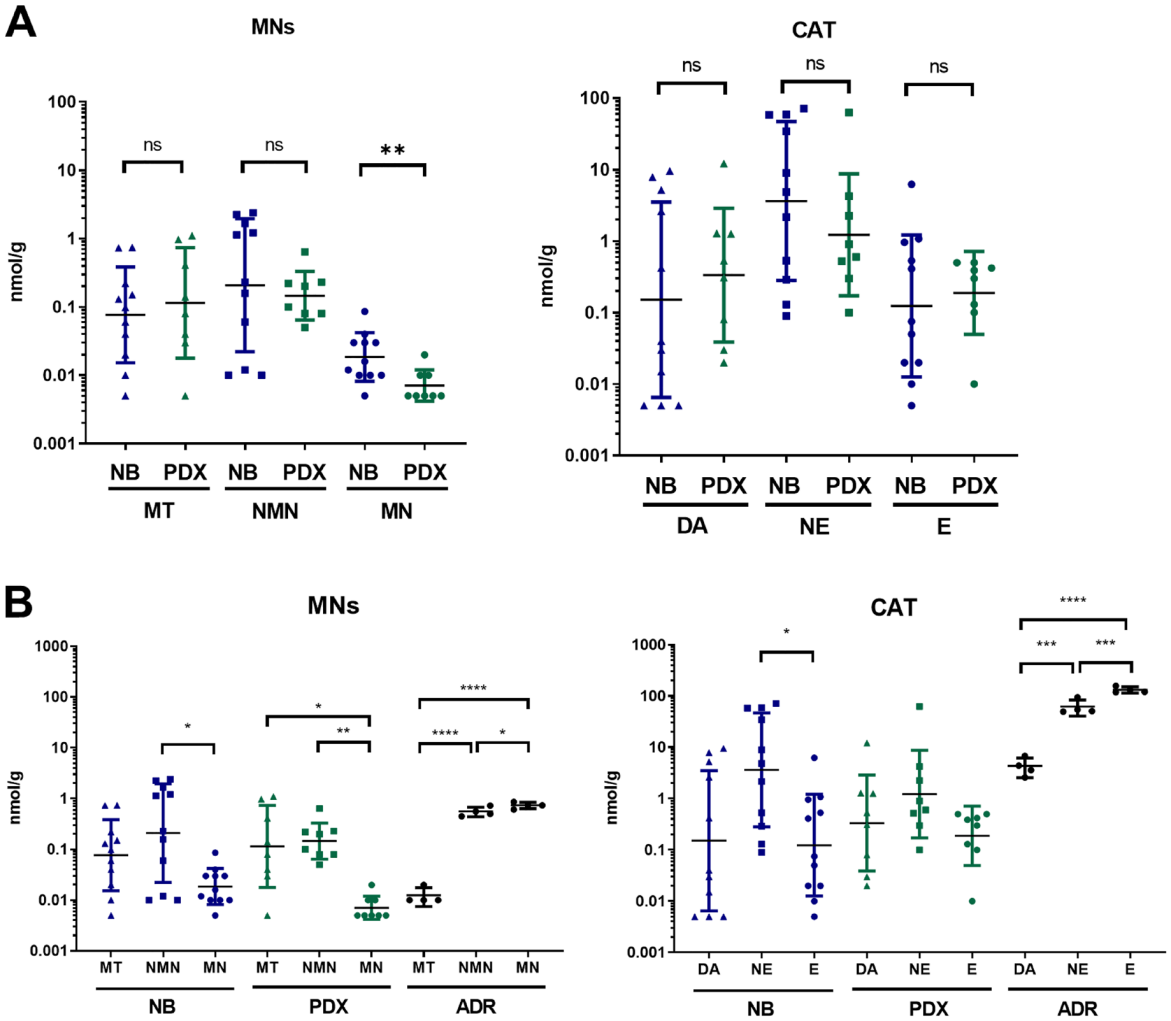
(**A**) Comparison of free MNs and CAT in tumor tissue of PDX mice (*n* = 8) and NB biopsy from patients (*n* = 11). Individual values (nmol/g) and geo mean ± geo SD are plotted on a logarithmic scale. Values under our limit of quantification were set at 0.005 nmol/g which represent half of the lower limit of quantification (LLOQ). Mann Whitney test: ^**^ *p* = 0.0078; (**B**) Tissue concentration of free MNs and CAT in NB primary tumors (*n* = 11), NB-PDX (*n* = 8) compared with adrenal glands (ADR) of control mice (*n* = 4). Kruskal-Wallis multiple comparison test for NB and NB-PDX, Ordinary one-way ANOVA for ADR: ^****^ *p* ≤ 0.0001, ^***^ *p* ≤ 0.0005, ^**^ *p* ≤ 0.005, ^*^ *p* ≤ 0.05, ns comparison are not shown (*p* > 0.05).

The intratumoral levels of CAT and MNs were then quantified in NB-PDX and NB biopsies (indicated in Table 1 and in Supplementary Table 2). Metabolites levels were present in low amounts in most of the tissues tested with concentrations close to the limit of quantification (0.01 nmol/g of tissue) for several samples and particularly for MN in NB-PDX. We detected comparable levels of each metabolite between both tumor groups without significant differences (Figure 3A), except for MN levels (geo mean at 0.02 and 0.01 nmol/g for respectively NB and NB-PDX for which most of the values were close or under the limit of quantification). We then compared MNs and CAT intratumoral profiles of NB primary tumors and NB-PDX relative to murine adrenal controls, as human adrenal samples were not available for this analysis. In the adrenal glands, the major forms of metabolites are E, NE, MN and NMN, while DA the early product of catecholamine metabolite synthesis and MT represent minor forms (Figure 3B). Interestingly, a profile distinct from the adrenal controls was observed in NB and in NB-PDX tumors, as weaker amounts of intratumoral E and MN were detected relative to NE and NMN (geo mean in nmol/g: E: 0.12 and MN: 0.02 vs NE: 3.63 and NMN: 0.21 for NB; and E: 0.19 and MN: 0.01 vs NE: 1.23 and NMN: 0.15 for NB-PDX) (Figure 3B). We refer to this profile as noradrenergic since E and MN synthesis are strongly reduced in NB tissues. This is consistent with the plasma MNs profile observed in the plasma of patients and mice (Figure 2C).

### The noradrenergic phenotype of NB results from the downregulation of PNMT expression in NB

**Figure 4:**
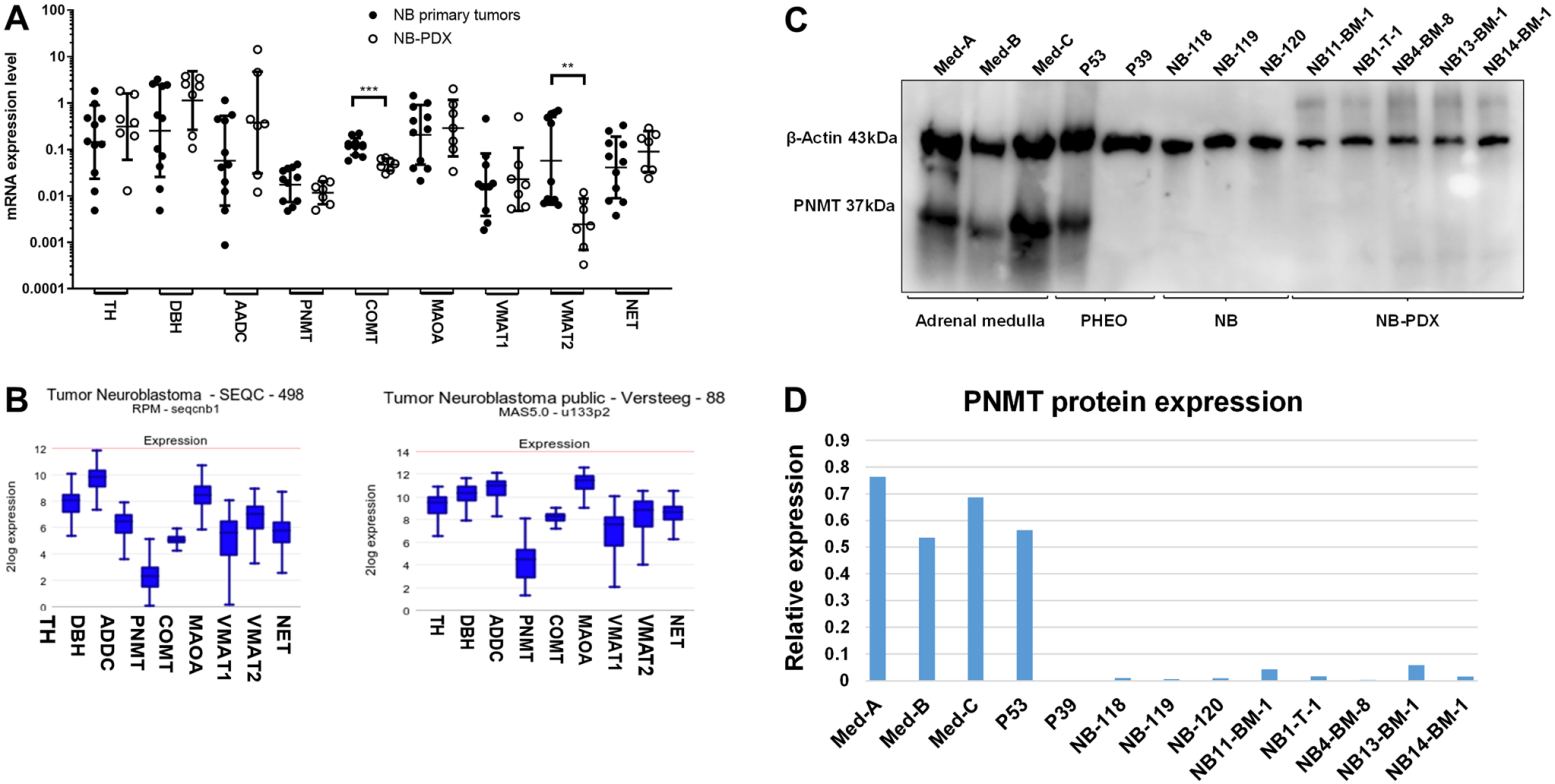
(**A**) mRNA expression levels of the main genes involved in CAT metabolism were analyzed by real-time qPCR in primary NB (*n* = 11) (black circles) and in NB-PDX (*n* = 7) (white circles). Data are plotted as mRNA expression level relative to the control genes TBP, GAPDH and EEIF1A1 with geo means ± geo SD (Mann Whitney test: COMT *p* = 0.0003, VMAT2 *p* = 0.0041, ns comparisons (*p* > 0.05) are not shown). qPCR analyses were performed in triplicats. (**B**) Illustration of the mRNA expression levels (normalized expression, in log2) of the indicated genes in two NB transcriptomic datasets analyzed by RNAseq (left) and microarrays (right). (**C**) Immunoblotting analysis of PNMT in representative tissues of three samples of adrenal gland tissue containing chromaffin cells, 2 PHEO, 3 NB and 5 NB-PDX. β-actin was used as loading control. (**D**) Densitometric quantification of immunoreactive band densities of Figure 4C using the Image J software (https://imagej.nih.gov/ij/download.html). The relative expression (PNMT/β-actin ratio) is plotted on the graph.

To validate further our murine NB-PDX models, we analyzed the expression levels of the main genes involved in catecholamine metabolism by real-time qPCR in NB-PDX and NB biopsies (indicated in Table 1 and Supplementary Table 2). Similar mRNA expression levels of TH, DBH, AADC, PNMT, MAOA, VMAT1 and NET were observed between human primary NB and NB-PDX, although a reduction of COMT and VMAT2 expression was measured in NB-PDX biopsies (Figure 4A). Using the R2: Genomics Analysis and Vizualization Platform (http://r2.amc.nl), we compared the expression levels of CAT genes between four datasets of NB primary tumors and a panel of adrenal glands (Supplementary Figure 2). TH was not significantly different between the two tissues, conversely to a strong upregulation for DBH, AADC, MAOA, VMAT2 and NET (*p* < 0.0001 for all comparison) in NB compared to adrenal glands (Supplementary Figure 2). On the other hand, COMT was downregulated in NB (*p* < 0.0001) without significant impact, considering that MNs are massively produced in the tumor (Supplementary Figure 2). In addition, PNMT, the enzyme that converts NE into E, was extensively downregulated in NB compared to normal tissue (*p* < 0.0001) (Supplementary Figure 2). This explains the noradrenergic profile observed in plasma and in NB tumor tissues.

PNMT protein expression was shown to be downregulated in NB cell lines [16]. Here we observed that PNMT mRNA expression level was detected at 3.7 to 73.6 fold lower level than other enzymes mRNA involved in catecholamine metabolism (Figure 4A). The scarce expression of PNMT compared with other CAT metabolism genes was confirmed by the analysis of the SEQC and Versteeg NB transcriptomic datasets [17, 18] using the R2 platform (Figure 4B). To confirm this data at the protein level, PNMT protein expression was analyzed in NB tissues from patients (Supplementary Table 2) and NB-PDX (Table 1) by immunoblotting. This revealed that PNMT protein was undetectable in the NB and NB-PDX tissues analyzed, as well as in one noradrenergic PHEO (P39, NE and E concentration in tissue: 14300 and 3 nmol/g respectively). As positive controls, PNMT was detected in an adrenergic PHEO (P53 with 737 and 901 nmol/g of NE and E) and in three samples of human adrenal medulla tissue containing chromaffin cells that produce PNMT (Figure 4C and 4D).

## DISCUSSION

Studies on NB biopsies suffers from a lack of material due to the low number of patients that undergo surgery procedure before treatment with chemotherapies and the needs of biopsy materials for pathology and genetic work up. In this report, we established mice NB xenograft models and validated their relevancy for the study of CAT metabolism and MNs as biomarker for NB detection. For this study, we took advantage of 4 NB-PDX models previously published by our group [13]. In addition, we developed four novel NB-PDX from metastatic NB cells of three patients and from the primary tumor of the patient NB-1, from which we already published a PDX derived from metastatic bone marrow cells [13]. Tumors raised in mice displayed histological phenotype of human primary NB and similar genomic profiles as their corresponding primary tumors. However, additional SCAs were identified in the NB-PDX, which may be explained by the fact that primary cells were cultivated for a short period *in vitro* before their subcutaneous injection in mice. In addition, the presence of chromosomal alterations differing from those identified in primary tumors were also described in disseminated NB cells in the bone marrow or using circulating tumoral DNA [19, 20].

**Figure 5:**
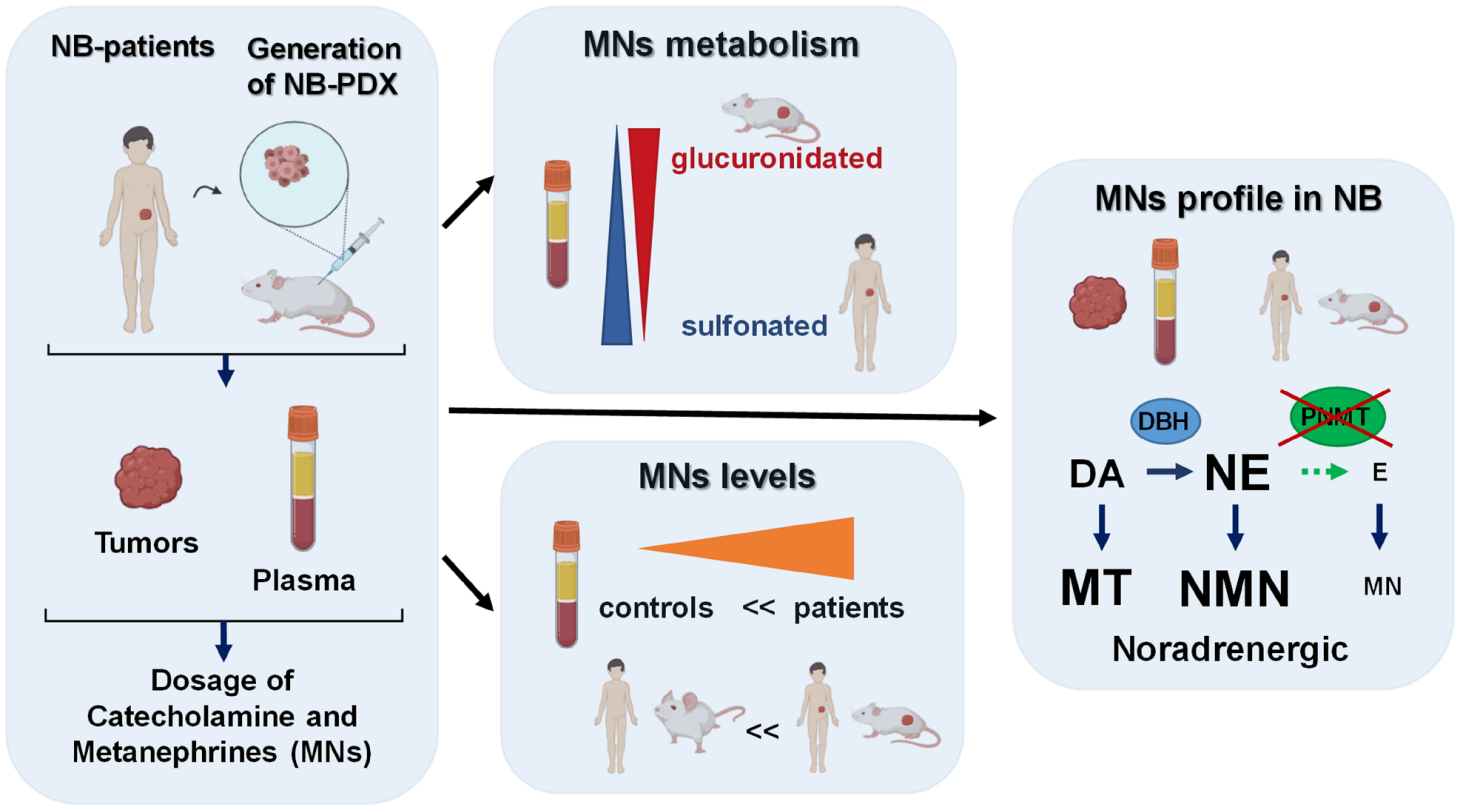
Graphical summary of our findings described in this report, as discussed in the results and discussion sections. This graphic was created using the software from Biorender (https://biorender.com/).

The novelty of this study is the demonstration that NB-PDX are not only genetically and phenotypically similar to human primary NB, but they also recapitulated CAT metabolism observed in NB patients (Figure 5). Indeed, we demonstrated that the levels of plasma MNs of NB-PDX-bearing mice were increased as observed in NB affected patients compared to controls. In addition, intratumoral CAT and MNs were found in equivalent proportions in NB-PDX and in NB primary tumors. This may be explained by the fact that the expression levels of the main genes involved in CAT synthesis and metabolism were also equivalent, except for COMT and VMAT2/SLC18A2 genes, which displayed lower expression levels in NB-PDX. These genes encode for the protein responsible for MNs production from CAT and for the monoamine transporter that internalizes CAT into neurosecretory vesicles in the cytoplasm, respectively. However, these differences are obviously of minor impact since MNs and CAT secretion profiles were conserved among the two types of tumors in both plasma and tumor biopsies.

Our data revealed that plasma MNs are mainly glucuronidated in mice, while sulfonation is a minor pathway, conversely to MNs in human plasma where sulfonation is by far the main process involved in MNs clearance in urine. Our observations are in accordance with a previous report describing in healthy rat a proportion of 63% of glucuronidated forms of NMN [21]. This implies that measurement of MNs in murine plasma requires a deconjugation step by glucuronidase to ensure a complete quantification (Figure 5).

Furthermore, we show in this report, that plasma and tumor tissues displayed a noradrenergic phenotype, as the synthesis of E and thus MN is limited in NB. This applies both in human and mouse NB models, reinforcing the validity of our NB-PDX models to mimic CAT metabolism found in patients. The noradrenergic catecholamine profile observed in NB is associated with the downregulation of PNMT expression, the enzyme responsible for E synthesis. Indeed, a very low amount of PNMT mRNA was detected in primary NB tumors and NB-PDX. Moreover, we demonstrated the lack of PNMT protein expression in NB tumor biopsies and in NB-PDX, as described only in NB cell lines in a previous report [16] (Figure 5). This is also in accordance with our former report showing that the downregulation of PNMT is responsible for the noradrenergic phenotype of a subset of PHEO compared with adrenergic PHEO [22]. In succinate dehydrogenase (SDH) and Von Hippel Lindau (VHL) related PHEO, PNMT mRNA downregulation has been demonstrated to arise from hypermethylation of the PNMT encoding gene [23, 24], similar mechanism may explain the noradrenergic phenotype of NB.

The limitations of this study are mainly due to the fact that our cohort of NB samples used for measurement of CAT and MNs in plasma, in tumor tissues and for the generation of NB-PDX are not overlapping. While NB biopsies used for intratumoral concentrations were obtained from high-risk stage L2 and M NB, our murine NB models were derived only from patients with high-risk stage M NB, which represents metastatic tumors associated with unfavorable prognosis. This is because less aggressive NB cells are not tumorigenic in athymic Swiss nude mice, as observed from previous work from our laboratory [13]. In addition, the primary NB cells were cultivated for a low number of passages *in vitro* before xenotransplantation, and NB-PDX harbor several additional SCA alterations as compared to NB tumors derived from the corresponding patients, which may affect their phenotypic characteristics or biological properties. Moreover, these PDX models may not fully reflect the exact NB microenvironment, which would have been reproduced in a more appropriate manner though orthotopic injection into the adrenal glands and/or by using small fragments of NB primary tumors which contains the additional stromal cells that represent key players in tumor progression [25–27]. However, despite these limitations, the NB-PDX models closely mimic the metabolism of catecholamine as observed in patients, regarding not only plasma levels of MNs, but also intratumoral CAT and MN concentrations, as well as mRNA expression levels of gene involved in CAT metabolism. Slight differences in gene expression were recorded for COMT and VMAT1, however without significant impact in terms of CAT and MNs production pattern. In addition, NB-PDX displayed a noradrenergic profile with reduced level of E and NM relative to NE and NMN, which was shown to correspond to the most frequent CAT profile in NB patients [16].

Plasma MNs quantification is on the rise to become the gold standard for NB diagnosis, since the test performance is better in terms of specificity and sensitivity than commonly used measurements of urinary CAT, VMA and HVA concentrations [11, 12, 28]. Plasma MNs level has been shown to correlate with tumor size in PHEO/PGL [29] and plasma MNs (totals forms) decrease within two days after PHEO/PGL resection [30]. Therefore, levels of MNs in mice models are also very likely to reflect the progression of tumor. Several models of NB in mice have been established [25, 27]. Apart from deciphering the molecular and genetic aspects of the tumors, as PNMT downregulation described in this report, NB preclinical models are also widely used for the assessment of new treatment strategies [25, 27]. However, the evaluation of the effects of therapeutics is essentially based on the observation of the tumor’s size through diverse monitoring methods (e.g., bioluminescence imaging, echography, micro-PET scan) requiring repeated anesthesia for a time-course follow-up. It would be interesting to evaluate if the measurement of plasma MNs may be relevant to monitor the therapeutic impact of drugs on NB-PDX models. Indeed, the low amount of plasma needed for MNs quantification by HPLC MS/MS (approx. 50 µl) is perfectly compatible with repeated blood collection in living mice without anesthesia. Thus, measuring plasma MNs at repeated time points (e.g., once a week) after the administration of drugs represents a less invasive method, which would allow implementing the principles of the 3Rs (Replacement, Reduction and Refinement) in animal experimentation.

To summarize, our NB-PDX model could represent a valuable tool for the study of catecholamine metabolism in NB and potentially for preclinical drug screening for NB therapy since previous studies have demonstrated a correlation between tumor size and metanephrine concentration. Blood testing of mice for metanephrine levels could therefore represent an option to monitor the growth of the tumors with limited impact on the animal welfare.

## MATERIALS AND METHODS

### NB, adrenal glands and PHEO/PGL tissues

PHEO/PGL samples were carefully chosen to be devoid of remaining healthy adrenal tissue by the surgeon or the pathologist. Similarly, healthy human adrenal glands were collected from the same procedure when healthy tissue was available and clearly distinct from the tumoral piece. The NB tumor material was collected from patients with stage L2 and M NB diagnosed at the Hemato-oncology Unit of the University Hospital of Lausanne (Switzerland) enrolled in the HR-NBL1 study from SIOPEN, after informed consent and in agreement with local institutional ethical regulations. This study was approved by the local ethics committee for the Canton de Vaud (Reference number: 2017-01865, 95/04 and 26/05). Of note the cohort of NB patients used for intratumoral CAT and MNs analyses and for the generation of NB-PDX are not overlapping.

NB primary cells were derived from high-risk stage M patients. Cells from the primary tumor of the NB1 patient were isolated as described [13]. Briefly, the tumor fragment was dissociated in a single-cell suspension by mechanical dissociation in phosphate-buffered saline containing 0.01 mg/ml collagenase II (Invitrogen, Carlsbad, CA, USA) and 0.1 mg/ml DNaseI (Roche Diagnostics, Basel, Switzerland) for 30 minutes at 37°C, followed by filtration through CellTricks (50 μm; Partek, Inc, St Louis, MO). NB primary cells derived from all other patients were isolated from involved BM. One volume (vol.) of BM was diluted with 3 vol. of Dubelco’s modified Eagle’s medium (D-MEM) (Life Technologies, Zug, Switzerland) and deposited on 2 vol. of Ficoll-Plaque Plus (GE Healthcare Europe) and centrifuged for 30 min at 1600 rpm. Then mononuclear cells were collected at the interphase, cells were washed twice in PBS and platted in neural basic medium (DMEM/F12 supplemented with penicillin/streptomycin, 2% B27 [Invitrogen], human recombinant basic fibroblast growth factor [FGF; 20 ng/ml; Peprotech, Rocky Hill, NJ], and EGF [20 ng/ml; Peprotech]). Once established, the purity of NB primary cells was then verified by performing anti-GD2 immunostaining. Primary NB cells NB1-T, NB1-BM and NB11-BM were also validated by IHC staining for Phox2b and SYP (Supplementary Figure 1B). The primary cells generated from the other patients were not evaluable by this method before implantation in mice.

### NB xenografts

All *in vivo* procedures were performed under the guidelines of the Swiss Animal Protection Ordinance and the Animal Experimentation Ordinance of the Swiss Federal Veterinary Office (FVO). Animal experimentation protocols were approved by the Swiss FVO (authorization number: VD2995 and VD3372). All reasonable efforts were made to reduce suffering, including anesthesia for painful procedures.

All PDX were generated using NB cells isolated from either a primary tumor (NB1-T) or from bone marrow aspirations and maintained *in vitro* for a limited number of passages (< 5). The only exception is the NB4-BM-8 PDX, which was generated from frozen cells dissociated from the seventh passage *in vivo* of the NB4-BM model (ie. NB4-BM-7). Primary NB cells (0.8 × 10^6^ to 2 × 10^6^) cells were suspended in 200 µl of Dulbecco modified Eagle medium DMEM (Invitrogen, Luzern, Switzerland) and BD Matrigel Basement Membrane matrix (1:1; BD Biosciences, San Diego, CA, USA) and implanted subcutaneously (s.c.) in the flanks of athymic Swiss nude mice (Charles River Laboratories, France). Tumor growth was followed up using calipers every 3 days. Mice were sacrificed once tumors reached a volume of approx. 900 mm3. NB-PDX were then maintained *in vivo* by serial subcutaneous transplantations. Tumor fragments were split into pieces for paraffin-embedded tissue formation, or collected in perchloric acid 0.1M for CAT and MNs quantification, or snap frozen in liquid nitrogen for protein or RNA extraction. NB xenograft fragments were also dissociated using the Tumor Dissociation kit Mouse (Miltenyi Biotec GmbH, Germany) according to manufacturer’s instructions, followed by filtration through CellTricks (50 μm; Partek, Inc, St Louis, MO, USA) and dissociated cells were frozen in cryo-preservative medium (50% DMEM, 40% FCS and 10% DMSO).

### Plasma collection

The control plasma were derived from 55 patients that were not affected by a NB, since the plasma MNs values were bellow upper reference limit. These patients were under 15 years of age, without previous MNs or CAT measurements request and none of them had MNs and CAT quantified ever since. For plasma from NB patients, a random set of 22 plasma was analyzed. The patients from which NB tissues were used to establish xenograft tumors (*n* = 7) were not part of this cohort of 22 patients. All blood samples were collected using a forearm venous cannula, with the patient kept supine for at least 15 min before sampling. Patients were instructed to fast and to abstain from caffeinated beverages for 24 hours before blood collection. All samples were collected onto ice and centrifuged within 30 min after puncture at 2500 g for 10 min at 4°C. Plasma was kept at −80°C until analysis. Total MNs and MT were desulfated using perchloric acid method or treated with sulfatase as described in a previous study [31]. Total and free MNs and MT were extracted on μElution plate and quantified by ultraperformance liquid chromatography-tandem mass spectrometry (UPLC-MS/MS) [32, 33]. The method used for free and total MNs quantification is identical after the deconjugation treatment applied to the sulfated MNs and MT [31].

For plasma collection from mice, immediately after mice sacrifice, blood was collected through heart puncture using 1 ml syringe (Omnifix-F, B.Braun, Melsungen, Germany) mounted with 25G5/8 needles (BD Microlance™ 3, Becton Dickinson, France). Blood was transferred in EDTA collection tubes BD Microtainer^®^ MAP (Becton Dickinson, USA). All samples were collected onto ice and centrifuged within 30 min after puncture at 2500 g for 10 min at 4°C. Plasma was kept at −80°C until analysis.

### CAT and MNs quantification

Tumor tissues and murine adrenal glands were disrupted in perchloric acid 0.1M and were sonicated using a Branson Sonifier 450 (Branson, Danbury, CT, USA) at full power for 30 seconds. CAT from tissue or plasma were extracted using activated alumina in 0.5 ml microcentrifuge tubes and quantified by ultraperformance liquid chromatography-tandem mass spectrometry (UPLC-MS/MS) [34]. MNs were extracted in homogenized tumor tissue and plasma and quantified as previously published [32, 33].

### Enzymatic hydrolysis of sulfates and glucuronides

Total MNs and MT were desulfated using perchloric acid method or treated with sulfatase as described in a previous study [31]. Glucuronides were removed enzymatically as follows: 10 µl of plasma samples were incubated with 90 µl of 10 mM ammonium acetate buffer pH 6.5 and 10 µl of beta-glucuronidase (Sigma-Aldrich, Buchs, Switzerland; previously dissolved in 2.5 M ammonium acetate buffer pH 5.5). Incubation time was 1 h at 37°C with agitation at 650 rpm. Samples were then diluted 2X in 10 mM ammonium acetate pH 6.5 and MNs were extracted as described above.

### RNA extraction and real-time qPCR

RNA extraction was performed using Trizol (Invitrogen, Luzern, Switzerland) or RNAeasy kit with DNaseI treatment according to manufacturer’s instructions (Qiagen, Hombrechtikon, Switzerland), and cDNA synthesis was performed using the PrimeScriptTM RT reagent Kit (Takara Bio Inc, Japan). Real time PCR assays were conducted in 384 wells using Sybergreen (Roche, Basel, Switzerland) with an Applied Biosystems 7900HT SDS (Thermo Fischer Scientific, Reinach, Switzerland). Negative controls were performed on the same amount of total RNA without adding the reverse transcriptase (NEC, no enzyme control). All primer pairs, chosen with the primer designing tool from the National Center for Biotechnology Information (NCBI) (Supplementary Table 3), were tested on a cDNA dilution series with efficiency comprised between 1.9 and 2.0. qPCR parameters were: 95°C 10 min, 40 cycles 95°C 15 sec. 60°C 1 min and melting curves were as expected in all cases. Normalization of gene expression was performed on the three reference genes (RG) TBP, GAPDH and EEIF1A1 using the ΔCt method with Ct_RG = (Ct_TBP+Ct_GAPDH+Ct_EEIF1A1)/3 and mRNA expression level = 2^-(Ct_Gene-Ct_RG)^.

### Immunoblotting

Adrenal glands remaining from PHEO surgery were used to obtain human adrenal medulla tissues. This was separated from the adrenal cortex using a scalpel by scraping off the brown interdigitated islets of chromaffin cells from adrenal glands. Tumor tissues and healthy parts of adrenal medulla were disrupted using a micro potter and lysed in a lysis buffer containing PBS with 0.5% triton X-100 and protease inhibitor (Complete™, Roche) to represent 20% w/v. Samples were then sonicated and clarified by a short centrifugation step of 2000 g for 30 seconds to discard the pellets. Samples were fractionated by SDS-PAGE under reducing conditions using precast gels (Bio-Rad, Reinach, Switzerland). Volumes loaded were 10 µl from a 20% wt/vol extract for biopsies and 5 µl from a 25 cm^2^ plate of confluent cells lysed in 200 µl of lysis buffer. Proteins were electroblotted onto nitrocellulose membrane and probed with antibodies. Immunoreactive bands were revealed by chemiluminescence assay (PerkinElmer, Schwerzenbach, Switzerland). Anti-PNMT polyclonal antibody raised in rabbit was purchased from Abcam (Lucerna-Chem AG, Luzern, Switzerland, ab167427) and anti-β-actin monoclonal antibody (AC-15) was purchased from Sigma-Aldrich (Buchs, Switzerland). Secondary HRP conjugated anti-mouse and anti-rabbit antibodies were from Bio-Rad (cat. number 170-6516 and 170-6515, respectively). Quantification of PNMT and β-actin immunorevelation was performed using the Image J software from NIH, USA.

### Immunohistochemistry and immunocytochemistry

The Hematoxylin and eosin (H&E) and immunohistochemistry (IHC) labeling were performed by the Lausanne Mouse Pathology Facility. IHC were performed on FFPE blocks of primary NB tumors, NB-PDX and cell pellets of the primary NB cells. IHC stainings were performed using anti-DBH Ab [9], anti-Phox2b (B11, # sc-376997, Santa Cruz Biotechnology, Dallas, USA), anti-TH (MAB318, MERCK KGaA, Darmstadt, Germany), and anti-SYP (NCL-L-SYPAP-299, Novocastra™, Leika Biosystems, Newcastle, UK) and polyclonal rabbit Ab anti-CD56/Ncam1 (14255-1-AP, Proteintech, USA).

For anti-GD2 immunocytochemistry, 200’000 cells were spin on cytospins with an Hettich (Hettich Zentrifugen, Tuttlingen, Germany) centrifuge at 700 rpm for 5 min. Slides were air-dried overnight, then fixed in 4% PBS-buffered paraformaldehyde for 10 min, washed in PBS, and incubated for 30 min at RT with monoclonal mouse anti-GD2 Ab (clone 7A4D8 [35]. After 3 washes in TBS 5 min., slides were incubated for 30 min at RT with rabbit anti-mouse antibody/AP (Dako, #D0314, Glostrup Denmark) diluted 1/30 in TBS, then with swine anti-rabbit Ig/AP (Dako, #D0306) diluted 1:20 in TBS for 30 min at RT, then with Fuchsin+ Substrate-Chromogene System (Dako, #K0625) for 10 min. After washing in running tape water for 5 min. slides were counterstained with hematoxylin (Sigma Aldrich, St Louis, MO, USA) and mounted with cover slip in buffered glycerine.

### SNP array analysis

DNA was extracted from fresh or frozen NB (for NB-11, NB-13, and NB-14) and frozen NB-PDX tissues, with a tumor cell content superior to 50%, on a EZ1 advanced XL DNA extractor using DNA Tissue Kit (Qiagen Hombrechtikon, Switzerland) and quantified on a NanoDrop ND-2000 spectrophotometer (Thermo Fisher Scientific, Inc.; Waltham, MA, USA). SNP array analysis was performed using the Affymetrix CytoScan HD platform (Thermo Fisher Scientific, Inc.; Waltham, MA, USA) according to the manufacturer’s protocol. Data analysis was performed using Chromosome analysis suite (ChAS) version 3.1.1.27 (r9436). CNA calling was performed by visual inspection.

### Statistics

The measurement data were explored statistically and graphically using Prism (v. 8.0, GraphPad Software, Inc. La Jolla, CA, US). Non-parametric Mann Whitney test was used to compare two different conditions. Multiple comparisons were performed by one-way Anova or Kruskal-Wallis test depending on data distribution. Only comparisons with a *p*-value < 0.05 were considered as statistically significant.

## SUPPLEMENTARY MATERIALS



## Abbreviations

MNs: metanephrines
NMN: normetanephrine
MN: metanephrine
MT: methoxytyramine
NB: neuroblastoma
CAT: catecholamine
E: epinephrine
NE: norepinephrine
DA: dopamine
PDX: patient-derived xenograft
PNMT: phenylethanolamine N-methyltransferase
DOPA: dihydroxyphenylalanine
TH: tyrosine hydroxylase
AADC: aromatic L-amino acid decarboxylase
VMAT1, 2: vesicular monoamine transporters 1 and 2
DBH: dopamine beta-hydroxylase
COMT: catechol O-methyltransferase
VMA: vanillylmandelic acid
HVA: homovanillic acid
PHEO: pheochromocytoma
PGL: paraganglioma
H&E: hematoxylin and eosin
IHC: Immunohistochemistry
Phox2b: Paired-like homeobox 2b
SYP: synaptophysin
CNA: copy number alterations
SNP: single-nucleotide polymorphism
SCA segmental chromosomal alterations; MAOA: monoamine oxidase A
NET: norepinephrine transporter
HR-NBL1: high risk neuroblastoma study 1
ChAS: chromosome analysis suite
