# Supplementary material for: The noradrenergic profile of plasma metanephrine in neuroblastoma patients is reproduced in xenograft mice models and arise from PNMT downregulation
Source: Oncotarget. 2021 Jan 5;12(1):49–60. doi: 10.18632/oncotarget.27858 (PMC7800772; doi:10.18632/oncotarget.27858)
Supplement: Supplementary file 1 [file oncotarget-12-49-s001.pdf]

The noradrenergic profile of plasma metanephrine in neuroblastoma patients is reproduced in xenograft mice models and arise from PNMT downregulation

SUPPLEMENTARY MATERIALS

Supplementary Table 1: List of SCA identified in NB tumors and NB-PDX derived from the corresponding patients

Patient ID	DNA	typSCA	atypSCA	PDX	typSCA	atypSCA
**NB1***	Primary T	1p-, 1q+, 17q+	4q+, 8q22-24.1+, 8q24.1-, 12q+, 14q-, 16q-, 19q-	**NB1-T-1**	1p-;1q+, 17q+, **3p-, 11q-**	4q+, 8q24.11+, 12q+, 14q-, 16q-, 19q-; **3q-, 5p-, 6q-, 7p+, 9p24-, 9q22.1+, 11q13.4-, 17p13.3-, 22q+**
**NB11**	T post chimio	1p36.33-, 2p24.3 ampl (MYCN)	1p21.3+, 1q31.2-, 1q42.12-; 14q32.2-	**NB11-BM-1**	1p36.33-, 2p24.3 ampl (MYCN)	**1p21.3-, 1p31.1-**,1q31.2-, 1q42.12-;
**NB13**	Primary T	1p36.33-, 2p25.3+, 2p24.3 ampl (MYCN), 17q12+	1p32.3+, 2q33.3-, 9q31.1+, 20p13-	**NB13-BM-1**	1p36.33-, 2p25.3+, 2p24.3ampl (MYCN), 17q12+	1p32.3+, 2q33.3-, 9q31.1+, 20p13-
**NB14**	Primary T	1p36-, 2p24.3p24.2 ampl (MYCN)	11p15-	**NB14-BM-1**	1p36-, p24.3p24.2 ampl (MYCN), **17q21.31+**	11p15-, **7p22.3+**

T, tumor; BM, bone marrow metastasis; typSCA, typical SCA; atyp, atypical SCA; +, gain; -, loss; ampl, amplification. Additional SCA observed in NB-PDX are indicated in bold. *Analyzed by CGH-array as previously published [13].

Supplementary Table 2: List of NB patient tumor samples and indication of the analyses carried out

Patient ID	CAT/MNs tumor	mRNA for CAT genes	PNMT protein (WB)
**NB-100**	✓	✓	n.a.
**NB-101**	✓	✓	n.a.
**NB-102**	✓	✓	n.a.
**NB-103**	✓	✓	n.a.
**NB-104**	n.a.	✓	n.a.
**NB-105**	n.a.	✓	n.a.
**NB-106**	n.a.	✓	n.a.
**NB-107**	n.a.	✓	n.a.
**NB-108**	n.a.	✓	n.a.
**NB-109**	n.a.	✓	n.a.
**NB-110**	n.a.	✓	n.a.
**NB-111**	✓	n.a.	n.a.
**NB-112**	✓	n.a.	n.a.
**NB-113**	✓	n.a.	n.a.
**NB-114**	✓	n.a.	n.a.
**NB-115**	✓	n.a.	n.a.
**NB-116**	✓	n.a.	n.a.
**NB-117**	✓	n.a.	n.a.
**NB-118**	n.a.	n.a.	✓
**NB-119**	n.a.	n.a.	✓
**NB-120**	n.a.	n.a.	✓

n.a.: not available.

Supplementary Table 3: Primers sequences used for qPCR

Gene	Protein encoded	Primers sequence (5′–3′)
**TH**	Tyrosine hydroxylase	**F**:GGC CGT GCA GCC CTA CCA AG
		**R**:ACG GAG AAG GGG CGC TGG AT
**DBH**	Dopamine beta-hydroxylase	**F**:GGC CGG GAG TGG GAG ATC GT
		**R**:TGT GGC CAG CTC CCG GTC TT
**AADC**	Aromatic-L-amino-acid decarboxylase	**F**:TCT GCC CTG CAG GAA GCC CT
		**R**:TTG TGG TCC CCA GGG TGG CA
**COMT**	Catecholamine-O-methyltransferase	**F**:GGA ATG TGG CCT GCT GCG GA
		**R**:CCG CGC ACG TGT GCT AGG AA
**PNMT**	Phenylethanolamine N-methyl-transferase	**F**:CTG CAG GGC TGG TGA TGC CC
		**R**:CGG GGG CCT TCA ACT GGA GC
**MAOA**	Monoamine oxydase A	**F**:GGG GGC TGC TAC ACG GCC TA
		**R**:TCC TGC CCA CGG GTT GAC GA
**NET**	Norepinephrine transporter	**F**:TGA TGG GGT TCA GGC CGG GT
		**R**:TTG GCC CAG GGC GGG AAG AT
**VMAT1**	Vesicule monoamine transporter 1	**F**:TGG GTC GGT GGC TGT GTT CC
		**R**:AGG CCA AGC CCT GCA TTG GG
**VMAT2**	Vesicule monoamine transporter 2	**F**:GCC TGG CAC GGA GGG CAA AT
		**R**:CCA CGC TGC TGA AGG ACC CG
**TBP**	Tata-box binding protein	**F**:GCC CGA AAC GCC GAA TAT A
		**R**:CGT GGC TCT CTT ATC CTC ATG A
**EEF1A1**	Eukaryotic transl. elong. factor 1 alpha 1	**F**:CTG AAC CAT CCA GGC CAA AT
		**R**:GCC GTG TGG CAA TCC AAT
**GAPDH**	Glyceraldehyde-3-phosphate dehydrogenase	**F**: F:CAT CCA TGA CAA CTT TGG TAT CGT
		**R**: R:CCA TCA CGC CAC AGT TTC C

F: forward, R: reverse.

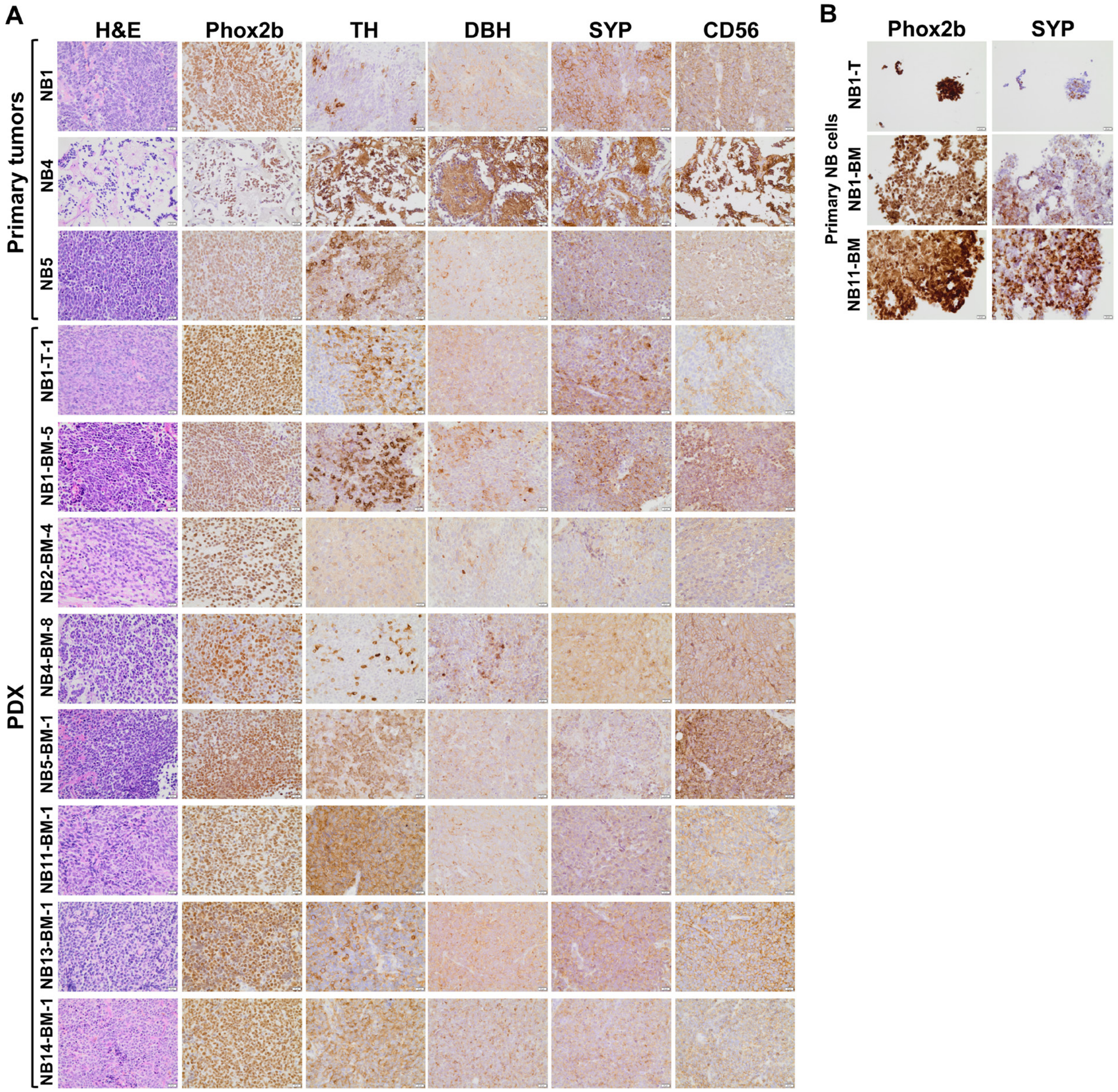

Supplementary Figure 1: Validation of the phenotype of NB-PDX and primary NB cells. (**A**) Representative images of H&E and IHC staining for Phox2b, TH, DBH, SYP and CD56 on NB primary tumors of patient NB1, NB4 and NB5 and the all NB-PDX investigated in this study. (**B**) Representative images of IHC staining for Phox2b and SYP on the primary NB cells NB1-T, NB1-BM and NB11-BM. The primary cells generated from the other patients were not evaluable by this method as all cells were used for the *in vivo* injection in mice. Scale bare: 20 m.

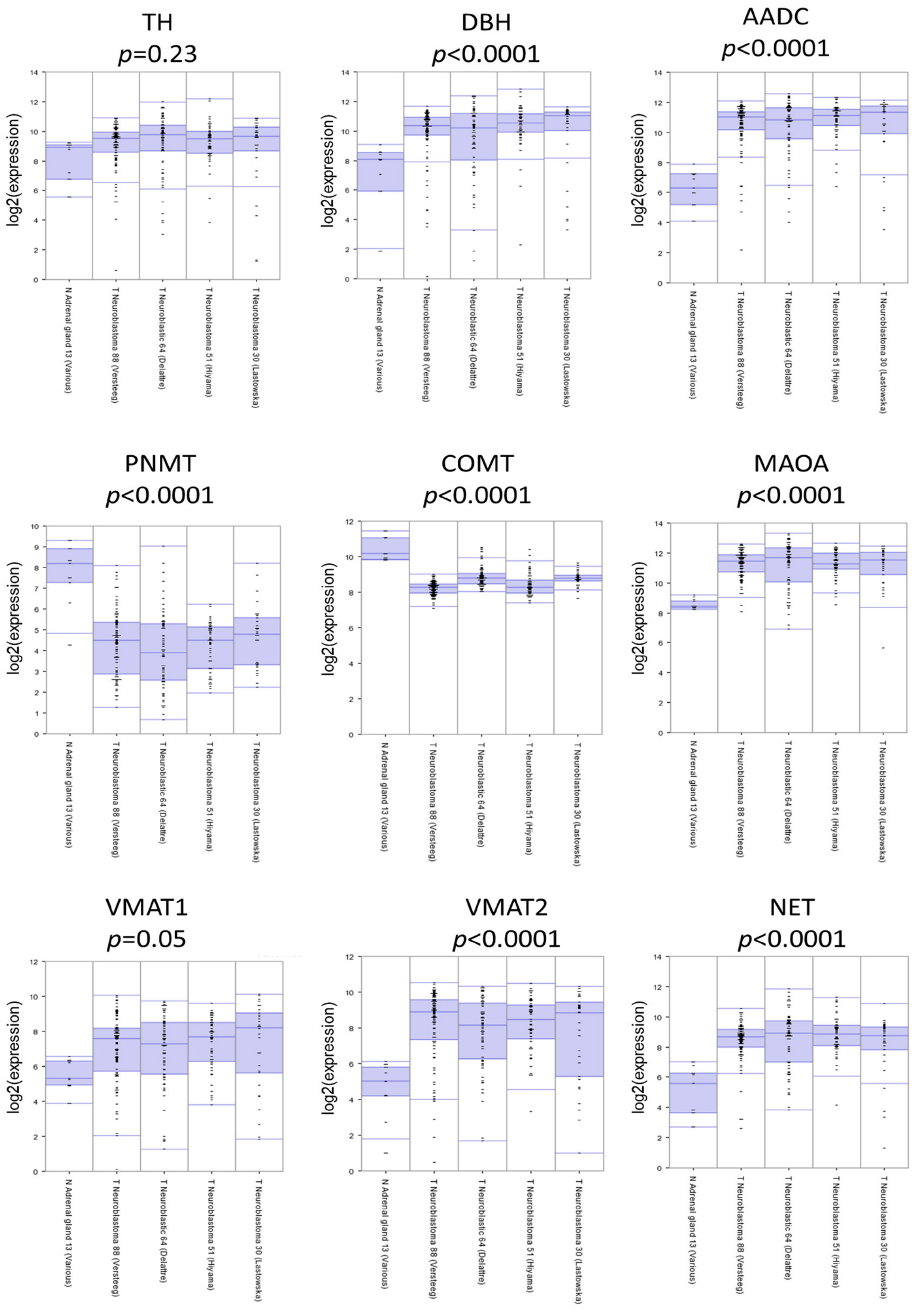

Supplementary Figure 2: Comparison of the expression levels (log2) of genes involved in CAT metabolism in normal adrenal gland (*n* = 13) and in 4 datasets of NB primary tumors. Versteeg, *n* = 88; Delattre, *n* = 64; Hiyama, *n* = 51; Lastowska, *n* = 30 using the R2: Genomics Analysis and Vizualization Platform (http://r2.amc.nl, MegaSampler analysis: Human Genome U133, Plus 2.0; MAS5.0 data normalization).
